# Case report: Clinical report of co-occurrence of pituitary adenoma and meningioma in the sellar region after meningioma treatment

**DOI:** 10.3389/fneur.2022.1042106

**Published:** 2022-12-06

**Authors:** Wang Lu, Yang Shengkai, Wang Yu, Li Aimin, Yan Shiwei, Xie Kang

**Affiliations:** ^1^Department of Neurosurgery, Affiliated Lianyungang Hospital of Xuzhou Medical University, Lianyungang, Jiangsu, China; ^2^Department of Neurosurgery, Jinzhou Medical University, Jinzhou, China; ^3^Department of Neurosurgery, Binhai County People's Hospital, Yancheng, Jiangsu, China

**Keywords:** meningioma, pituitary adenoma, sellar region, MRI, vision

## Abstract

The coexistence of meningioma and pituitary adenoma is very rare, especially in the same location after meningioma surgery. Here, we reported a case of coexisting meningioma and pituitary adenoma secondary to postoperative meningioma in the sellar region in a patient who had not received radiation therapy before the second surgery. A 61-year-old woman underwent craniotomy for tumor resection for sellar meningioma in 2017, and postoperative imaging showed no residual in the surgical area. In 2022, the patient had a history of decreased vision again. MRI showed the possibility of postoperative pituitary adenoma in the sellar region. The patient underwent endoscopic resection of the skull base lesion again. After surgery, the patient's visual symptoms improved. Histology of the sellar tumor showed both meningioma (meningeal epithelial type and WHO grade I) and pituitary adenoma in the same section. The coexistence of meningioma and pituitary adenoma is a very rare surgical entity. This report provides a theoretical basis for the selection of intracerebral tumor surgery and provides a diagnostic basis and treatment reference for patients diagnosed with meningioma and pituitary adenoma at the same time.

## Introduction

The sellar region is located in the central portion of the skull base, behind the posterior wall of the sphenoid sinus and between both cavernous sinuses, and is one of the locations with a high incidence of intracranial tumors. The sellar area is adjacent to the internal carotid artery, optic nerve, pituitary gland, hypothalamus, and other vital structures (1). A space-occupying lesion will cause compression symptoms, resulting in headaches, visual field defects or decreased vision, and endocrine abnormalities. In severe cases, coma may occur. The anatomical location of the pituitary tumor is located in the pituitary recess in the sella region of the skull base, which is connected with the hypothalamus and adjacent to the optic chiasm. Therefore, pituitary tumors are more common in the sellar region.

As one of the most common intracranial tumors, meningioma originates from the intracranial meninges, with an incidence of about 15–25% (2). Generally, meningioma mainly originates from arachnoid villi aberrations, which often occur in venous sinuses. Patients with meningioma in the sellar region are only 2.1% (3). Among the pathogenic factors of meningioma, radiation is a recognized carcinogenic factor, and its specific mechanism is unclear. In the absence of radiation stimulation, secondary meningioma after intracranial tumor surgery is very rare.

This study reported a rare vision loss 4 years after meningioma surgery. MRI examination revealed tissue lesions in the sellar region. The patient underwent minimally invasive neurosurgery. The postoperative tumor pathology results showed meningiomas and pituitary adenomas in the intracranial sellar region. The pathological results suggest that the tumors in the sellar tissue are also diverse, not as simple as a single tumor. Therefore, when dealing with this type of disease, it is necessary to comprehensively consider and combine neuroendoscopy, neuronavigation technology, and microsurgery technology to select the optimal treatment plan, so as to accurately locate the tumor location and meet the requirements of total tumor resection and subtotal tumor resection. This further reduces the incidence of postoperative complications.

## Case presentation

**Figure 1:**
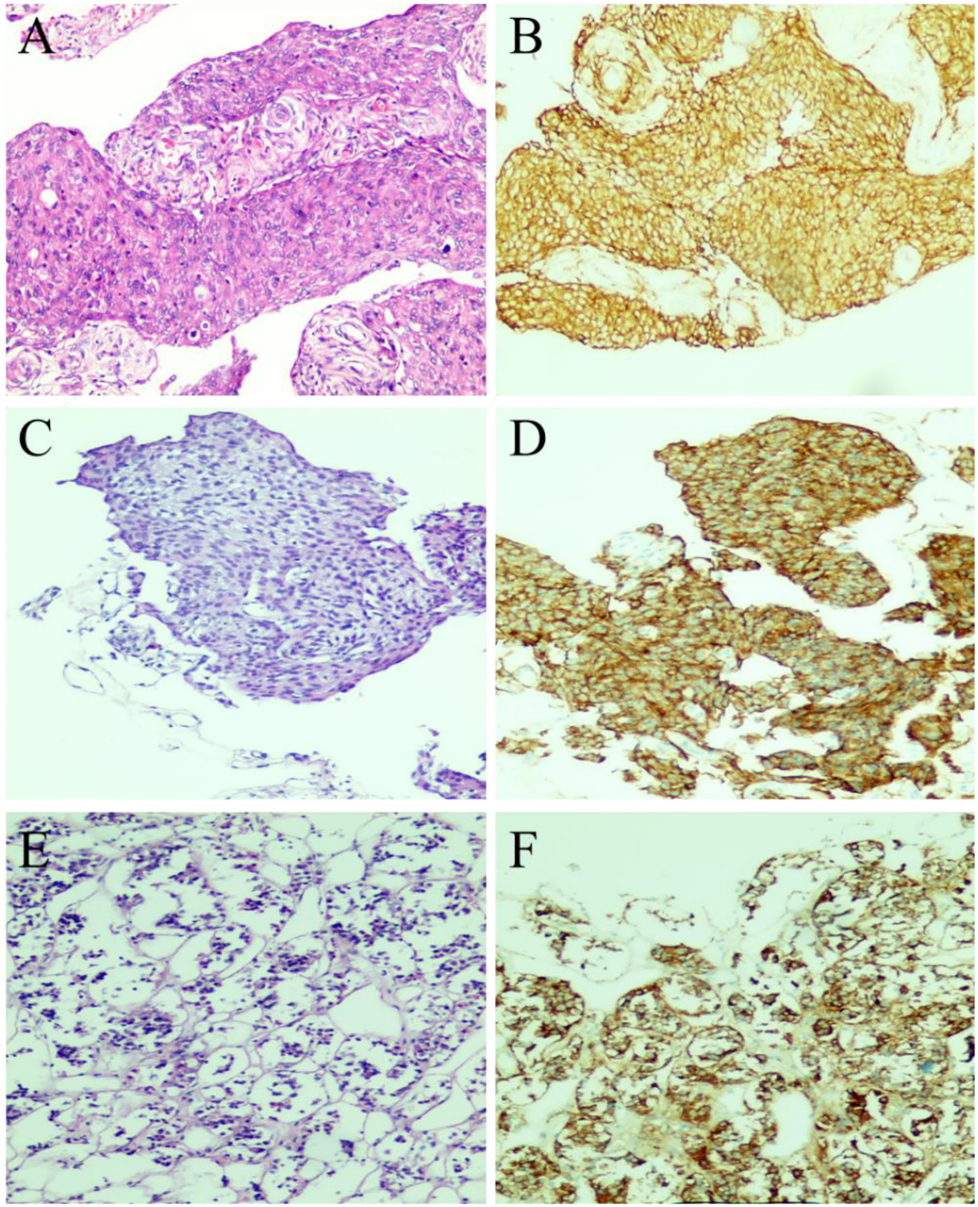
Pathological examination results of tumor resection. **(A)** The first surgical pathology results in 2017: meningioma (meningeal epithelial type, WHOI grade). **(B)** Immunohistochemical results of meningioma in 2017: GFAP(–), Ki-67(1%+), S-100(–), PR(3+), EMA(3+), CK7(–). **(C,D)** Postoperative histological examination of the tumor in the sellar region showed meningioma (meningeal epithelial type and WHO I grade), and the immunohistochemical results were as follows: GFAP(–), Ki-67(1%+), S-100(–), PR(2+), EMA(3+). **(E,F)** The immunohistochemical results of recurrent pituitary adenoma in the sellar region were as follows: EMA (3+), Syn (2+), Ki-67 (1%+), and PR (2+).

**Figure 2:**
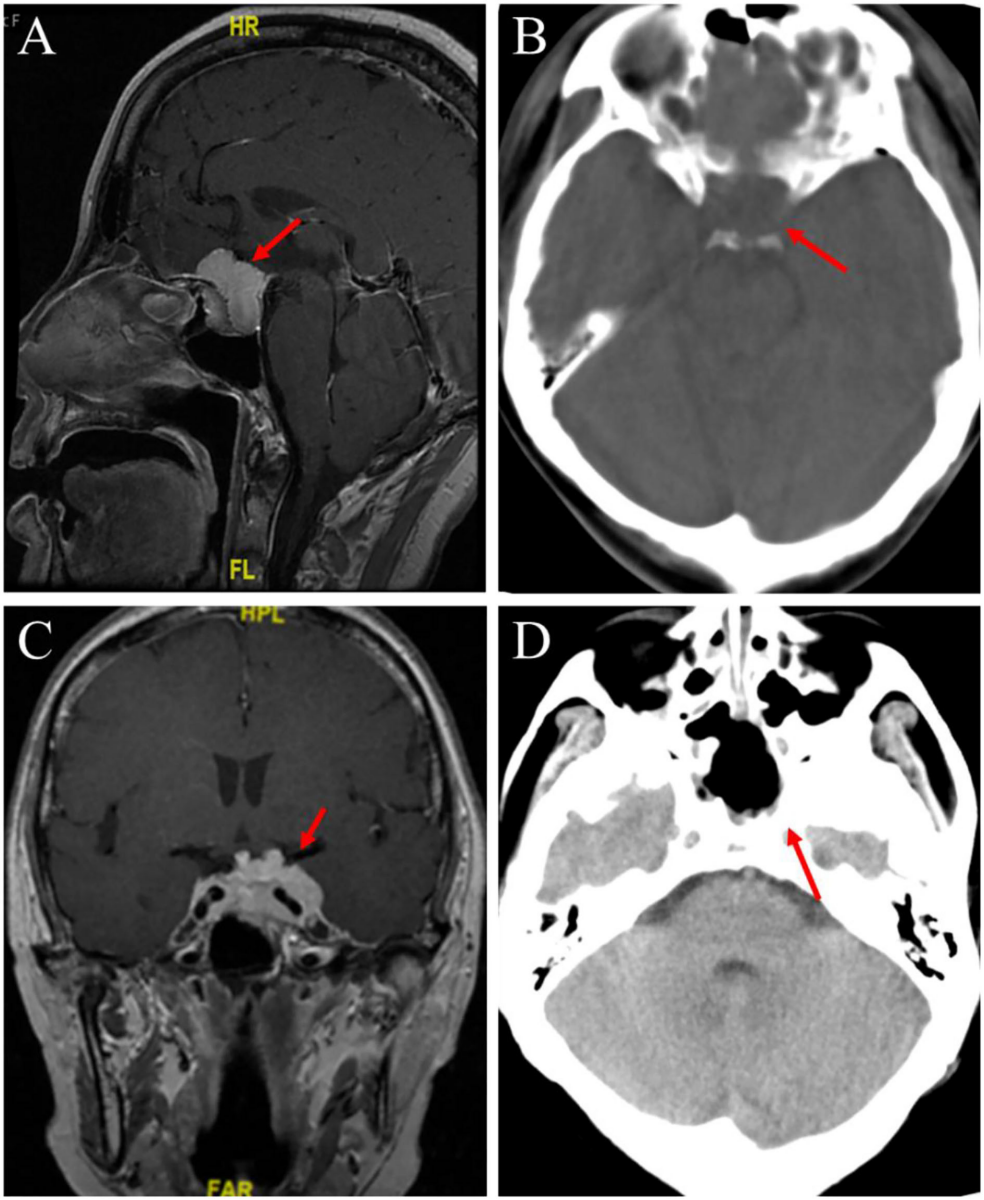
Preoperative head MRI and postoperative head CT examination were performed. **(A)** An enhanced MRI scan of the head before the first operation, the red arrow points to the pituitary fossa mass. **(B)** In the first postoperative CT scan, the red arrow points to the complete resection of the meningioma tumor. **(C)** T1-weighted enhanced coronal image before the second operation, the red arrow points to the same shadow of the newly formed T1T2 signal in the post-saddle region. **(D)** In the second postoperative CT scan, the red arrow points to the complete resection of the sellar mass.

A 61-year-old female patient underwent an MRI examination in 2017 due to the progressive decline of binocular vision, which showed intracranial sellar space-occupying lesions, and underwent craniotomy for tumor resection. The postoperative pathological results showed meningioma, meningeal epithelial type, and WHO I type (Figures 1A,B). Symptoms improved at discharge, and no postoperative radiation therapy was given. The patient recently presented with symptoms of decreased visual acuity again without other neurological symptoms. The MRI report showed that the operative area was enhanced with flaky patches (Figure 2C), possibly a pituitary tumor. After communicating with the patient, endoscopic skull base lesion resection was performed again on 20 April 2022. The patient's visual acuity improved after surgery, and the neurological examination was normal at the time of discharge (Figure 2D). The pathological results of the tumor after the second operation showed meningioma, meningeal epithelial type, WHO grade I (Figures 1C,D); pituitary tumor, some pituitary adenomas, and some meningiomas (Figures 1E,F).

### The plasma level of the pituitary hormone

This patient's clinical manifestation of the adenoma was a non-functional adenoma, which only shows that the tumor compresses the optic nerve and causes vision loss. The detection of hormone level in patients before the second operation, thyroid-stimulating hormone (TSH): 2.03 mIU/L (reference range: 0.56–5.91 mIU/L); Free T3 (FT3): 4.63 pmol/L (reference range: 3.8–6.47 pmol/L); Free T4 (FT4): 6.45 pmol/L (reference range: 7.9–17 pmol/L); T3: 1.52 nmol/L (reference range: 1.34–2.73 nmol/L); T4: 83.24 nmol/L (reference range: 78.4–157.4 nmol/L); luteinizing hormone (LH): 6.82 mIU/ml (reference range: 10.87–58.64 mIU/ml); follicle-stimulating hormone (FSH): 26.97 mIU/ml (reference range for postmenopausal women: 16.74–113.59 mIU/ml); pituitary prolactin (PRL): 34.29 ng/ml (reference range of postmenopausal women is 2.74–19.64 ng/ml); growth hormone (GH): 0.07 ng/ml (reference range: 0.01–3.607 ng/ml); and cortisol (Cor): 97.830 μg/L (reference range: 60.2–184 μg/L).

### Neurological examination

The patient has a clear mind, good mental state, equal and equal round pupils on both sides, with a diameter of about 3 mm, sensitivity to light reflex, normal eye movement, and poor vision in both eyes, the results of Octopus visual field examination showed that the peripheral visual field of the left eye was significantly defective, and the temporal visual field of the right eye was slightly defective. The visual acuity of the right eye was 0.6, the visual acuity of the left eye was 0.3, and the visual acuity of the left eye decreased significantly, and no abnormality in the nasal cavity and external auditory canal. The forehead lines, nose, and lip grooves were symmetrical, and the tongue was in the middle. The forehead and face feel was normal, and the mouth feel was strong, the Weber test was in the middle, and the corneal reflex, pharyngeal reflex, and head–eye reflex were normal. The shoulders were symmetrical on both sides. The neck was soft, without resistance. The muscle strength of both limbs was Grade V, the muscle tension was normal, the deep and shallow senses of the limbs were normal, the superficial reflex was normal, and the tendon reflex was not hyperfunction. Bilateral pathological signs were not elicited.

### Cranial impact examination

On 27 March 2017, the preoperative enhanced MRI scan of the patient's head showed that a 2.99 × 1.75 cm enhanced focus was found in the pituitary fossa, with blood vessels running inside and optic nerve compression (Figure 2A).

On 3 April 2017, postoperative CT of the patient showed that the part of the frontal bone was absent, no obvious abnormality was found in the brain parenchyma and ventricular system, and the midline structure of the brain was in the middle (Figure 2B).

On 19 April 2022, a plain scan and enhanced MRI of the patient's head before the operation showed that after the operation in the sellar region, the size of the tumor was about 2.8 × 4.0 cm, lumps and other T1–T2 signal mass shadows could be seen in the operation area. The tumor body wrapped around the internal carotid artery on enhanced scan and pushed upward to press the optic chiasma (Figure 2C).

On 21 April 2022, there was a slightly high-density strip shadow under the skull plate, no obvious abnormality was found in the ventricular system, and the midline structure of the brain was in the middle (Figure 2D).

### Treatment and prognosis

The patients should complete preoperative vital signs, laboratory, imaging-related examinations, and determine the tumor location and operation mode. Selected a 30-degree endoscope to place into the nasal cavity, exposed the opening of the sphenoid sinus, and cut the nasal septum mucosa at the middle turbinate to fully expose the surgical field. The surgical area showed the tumor was flesh red, hard, and rich in blood supply. The tumor should be removed in blocks. The tumor removal sequence was first at the saddle bottom, then at both sides, and then at the middle. The tumor showed invasive growth. The tumor near the internal carotid artery was tightly adhered and was retained in a minimal amount.

After the operation, both internal carotid arteries were well-protected, and the artificial meninges at the sellar floor were completely repaired. The patient was discharged from the hospital, the patient had a clear mind, no apparent blood exudation in the nasal cavity slightly decreased sense of smell, equal large and equal round pupils on both sides, light sensitivity, no apparent decline in vision, and no apparent neurological impairment symptoms left.

After discharge, we regularly went to the outpatient department of our hospital for reexamination. Furthermore, the patient's vision does not continue to decline, his olfactory sense returns to normal, and no tumor growth is found in the postoperative reexamination imaging. Considering the relatively short recurrence interval of the tumor and the minimal amount of residual tumor near the carotid artery during the operation, we were now receiving gamma knife intracranial directional radiotherapy.

## Discussion

Whether pituitary adenomas and meningiomas can interact and transform is unclear. The preferred diagnosis of single sellar lesions is a pituitary adenoma. Studies have found that 10–15% of patients with intracranial tumors were diagnosed with pituitary adenomas by autopsy, and 23% were diagnosed with meningiomas by MRI (4). The single incidence is still low, which shows that the coexistence of pituitary tumors and meningiomas is rare. It has been reported that pituitary adenomas after radiotherapy can coexist with meningiomas (5, 6). However, Partington and Davis (7) reported that this phenomenon could also occur in patients who have not received radiotherapy. Doubts remain about whether two distinct treatment outcomes might indicate an association between the two tumors. Whether pituitary adenomas may increase the probability of secondary meningioma and whether pituitary adenomas and meningiomas in the sellar region can transform into each other or evolve has no scientific theoretical support.

However, other scholars believe pituitary tumors, as endocrine tumors, are closely related to their clinical manifestations and hormone secretion. The increase in endogenous hormones that can cause tumors of different tissue types to interact with each other is unknown. Wiemels et al. (8) explained that prolactin is essential in stimulating meningioma growth. Prevedello et al. (9) studies have shown that the most common hormone to stimulate the growth of meningioma is growth hormone. Numerous studies have shown that hormones secreted by the pituitary gland may be the key to inducing abnormal proliferation of meningeal cells to form meningiomas. However, this theory has not been scientifically proven.

The pathogenesis of meningioma may be closely related to gene mutation. The mutation or deletion of the tumor suppressor factor (NF2) gene, which most often occurs on chromosome 22, can be found in more than 50% of sporadic meningioma patients. With the rapid development of new sequencing technology, some mutation genes commonly found in meningiomas, such as SMO, AKT1, PIK3CA, KLF4, BAP1, POLR2, SMARCE1, and TRAF7. SMO, AKT1, and PIK3CA mutations are specific mutations in anterior skull base meningiomas, while KLF4 mutations are highly expressed in secretory meningiomas, and BAP1 mutations are common in rhabdomyoma meningiomas (10). The primary pathological mechanism of pituitary adenoma is epigenetic mutation leading to cell aberration. A large number of studies have found that somatic mutations (GNAS, USP8, GPR101) and germline mutations (MEN1, cyclin-dependent kinase inhibitor gene, AIP, DICER1, SDHx, and GPR101) are closely related to the pathogenesis of different clinical types of pituitary tumors (11). Therefore, it is significant to study the genes related to pituitary adenoma and meningioma to prevent and treat both.

Considering the high recurrence rate and initial location of the tumor at the same site as the patient, “SMO mutation” may exist in this case. SMO mutation is mainly found in anterior skull base meningioma, which is obviously abnormal with the normal meningioma-prone site. It is a vital signal converter in the sonic hedgehog (SHH) signal pathway and is inseparable from embryonic development and tumor occurrence. It plays a vital role in the process of intracranial lesions in patients with aggressive, unresectable, and highly recurrent meningioma. Unfortunately, due to the immaturity of advanced theoretical knowledge and the lack of detection technology, the patient failed to carry out relevant gene detection to determine the existence of SMO gene mutation in the patient's cells (13, 14).

With the incidence of pituitary tumors, there is still no effective drug treatment. Therefore, surgery is the best choice for treatment. Despite this, the recurrence rate of patients within 10 years is still as high as 7–12% (10, 11), and the prognosis is not as expected. Therefore, a comprehensive and in-depth understanding of the molecular biological characteristics of different types of pituitary adenomas is conducive to improving the prognosis of pituitary adenomas (12). Meanwhile, the current international guidelines guide the optimal treatment of meningioma is also surgery, but whether adjuvant radiotherapy or surgery can be selected for atypical or anaplastic meningioma. However, for skull base meningiomas surrounding blood vessels or neural structures, as well as refractory and recurrent meningiomas, repeated surgery is still the best choice.

MRI is commonly used in the diagnosis of sellar tumors, but in the process of clinical diagnosis, it is found that the imaging manifestations of pituitary adenomas and intrasellar meningiomas are very similar, and MRI cannot differentiate them before surgery. Therefore, intraoperative histopathological examination is an essential step in identifying tumor attributes. The MRI examination of the patient reported in this study showed the state of the sellar region, and the operation area showed a mass shadow with T1 and other T2 signals. The enhanced scan showed uniform enhancement, which seemed to be a single tumor. However, intraoperative histopathological examination confirmed the coexistence of pituitary adenoma and meningioma, and the pathological indicators provided a theoretical basis for postoperative treatment.

After meningioma surgery, pituitary adenoma and meningioma coexist in the same sellar region, making the operation more difficult. According to the experience of senior doctors, when the pituitary adenoma and meningioma are adjacent to each other, a single pterional approach or endoscopic single intranasal transsphenoidal approach can be used for one-time surgical resection. Compared with conventional surgery, this approach significantly increases the risk of surgery and poor prognosis. Therefore, perfect preoperative examinations, a complete understanding of the specific conditions of the location and function of intracranial tumors, and an assessment of the patient's general condition are essential prerequisites for formulating appropriate surgical approaches and avoiding severe complications.

## Conclusion

The imaging manifestations of sellar tumors are similar, and a definitive diagnosis cannot be made preoperatively. Therefore, intraoperative histopathological examination is crucial, which can provide a theoretical basis for the subsequent treatment.

## Data availability statement

The original contributions presented in the study are included in the article/supplementary material, further inquiries can be directed to the corresponding authors.

## Ethics statement

The studies involving human participants were reviewed and approved by Affiliated Lianyungang Hospital of Xuzhou Medical University. The patients/participants provided their written informed consent to participate in this study. Written informed consent was obtained from the individual(s) for the publication of any potentially identifiable images or data included in this article.

## Author contributions

WL, YShe, and WY: data analysis and interpretation. WL, YShe, WY, and LA: drafting of the manuscript. LA: acquisition of data. YShi: critical revision of the manuscript. XK and YShi: study concept and design. XK: study supervision. All authors read and approved the final manuscript.

## Funding

This study was supported by the Organization Department of Jiangsu Province and Jiangsu Provincial Health Commission (ZDA2020018).

## Conflict of interest

The authors declare that the research was conducted in the absence of any commercial or financial relationships that could be construed as a potential conflict of interest.

## Publisher's note

All claims expressed in this article are solely those of the authors and do not necessarily represent those of their affiliated organizations, or those of the publisher, the editors and the reviewers. Any product that may be evaluated in this article, or claim that may be made by its manufacturer, is not guaranteed or endorsed by the publisher.
